# Supplementary material for: Three-Dimensional Modeling of CpG DNA Binding with Matrix Lumican Shows Leucine-Rich Repeat Motif Involvement as in TLR9-CpG DNA Interactions
Source: Int J Mol Sci. 2023 Oct 8;24(19):14990. doi: 10.3390/ijms241914990 (PMC10573802; doi:10.3390/ijms241914990)
Supplement: Supplementary file 1 [file ijms-24-14990-s001.zip › ijms-2596716-supplementary.pdf]

Supplementary Information for

Three-dimensional modeling of CpG DNA binding with matrix lumican shows leucine-rich repeat motif involvement as in TLR9-CpG DNA interactions

Tansol Choi[1], George Maiti[1*] and Shukti Chakravarti[1,2*]

[1] Department of Ophthalmology, NYU Grossman School of Medicine, New York, NY 10016, USA

[2] Department of Pathology, NYU Grossman School of Medicine, New York, NY 10016, USA

This supplementary document includes the following figures:
Figure.S1 to S5

```
LUM_MOUSE      3 VCAFSLALALVGSVSGQYYDYDIPLFMYGQISPNCAPECNCPHSYPTAMY  52
TLR9_MOUSE    19 VLAETLALG------------TLPAFLPCELKPHGLVDCNW---------  47
                 |.|.:|||.            .:|.|:..::.|:...:||.

                                             LRR1              LRR2
LUM_MOUSE     53 CDDLKLKSVPMVP-----PGIKYLYLRNNQIDHIDEKAFENVTDLQWLIL  97
                                             LRR1             LRR2
TLR9_MOUSE    48 ---LFLKSVPRFSAAASCSNITRLSLISNRIHHLHNSDFVHLSNLRQLNL  94
                    |.|||||...     ..|..|.|.:|:|.|:....|.::::|:.|.|

                           LRR2                        LRR3
LUM_MOUSE     98 DHNLLE----------NSKIKGKVFSKLKQLKKLHINYNNLTESVGPLPK  137
                       LRR2                            LRR3
TLR9_MOUSE    95 KWNCPPTGLSPLHFSCHMTIEPRTFLAMRTLEELNLSYNGIT-TVPRLPS  143
                 ..|...          :..|:.:.|..::.|::|:::||.:| :|..||.

                         LRR4                       LRR5
LUM_MOUSE    138 SLQDLQLTNNKISKL--GSFDGLVNLTFIYLQHNQLKEDAVSASLK----  181
                 LRR3         LRR4                  LRR5
TLR9_MOUSE   144 SLVNLSLSHTNILVLDANSLAGLYSLRVLFMDGNCYYKNPCTGAVKVTPG  193
                 ||.:|.|::..|..|  .|..||.:|..:::..|...::..:.::|

                                LRR6
LUM_MOUSE    182 ---GLKSLEYLDLSFNQMSKLPAGLPTSL--------------------L  208
                               LRR6                   LRR7
TLR9_MOUSE   194 ALLGLSNLTHLSLKYNNLTKVPRQLPPSLEYLLVSYNLIVKLGPEDLANL  243
                    ||.:|.:|.|.:|.::|:|..||.||                    |

                                                               LRR7
LUM_MOUSE    209 T--------------------------------------------LYLDN  214
                           LRR8                              LRR9
TLR9_MOUSE   244 TSLRVLDVGGNCRRCDHAPNPCIECGQKSLHLHPETFHHLSHLEGLVLKD  293
                 |                                            |.|.:

                      LRR7               LRR8                 LRR9
LUM_MOUSE    215 NKISNIPDEYFKRFTGLQYLRLSHNELADSGVPGNSF-NISSLLELDLSY  263
                     LRR9                 LRR10               LRR11
TLR9_MOUSE   294 SSLHTLNSSWFQGLVNLSVLDLSENFLYESITHTNAFQNLTRLRKLNLSF  343
                 :.:..:...:|:....|..|.||.|.|.:|....|:| |::.|.:|:||:

                     LRR9                 LRR10                LRR11
LUM_MOUSE    264 NKLKSIPTVNENLENYYLEVNELEKFDVKS-FCKILGPLS------YSKI  306
                    LRR11                  LRR12              LRR13
TLR9_MOUSE   344 NYRKKVSFARLHLASSFKNLVSLQELNMNGIFFRLLNKYTLRWLADLPKL  393
                 |..|.:.....:|.:.:..:..|::.::.. |.::|...:      ..|:

                      LRR11
LUM_MOUSE    307 KHLRLDGNPLTQSSL  321
                      LRR13
TLR9_MOUSE   394 HTLHLQMNFINQAQL  408
                 ..|.|..|.:.|:.|
```

| Identical : Semi-conservative . Least-conservative

Figure S1: <u>Pair-wise sequence alignment between lumican and TLR9 ECD</u>. The Clustal Omega pairwise sequence alignment between lumican and TLR9 ECD indicates 39.8% and 22.2% sequence similarity and identity, respectively.

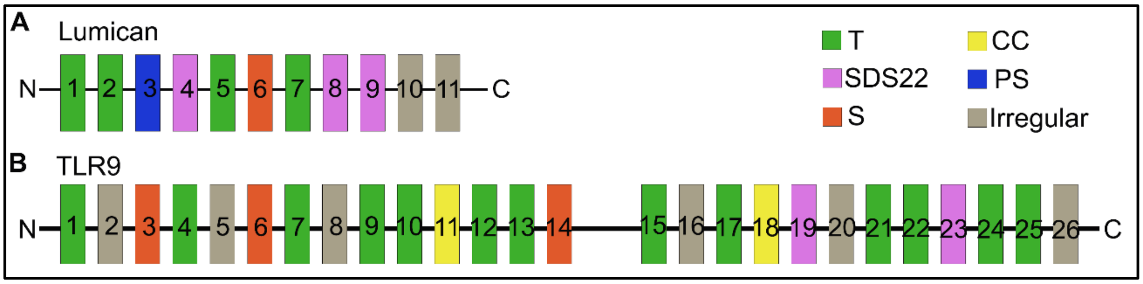

Figure S2: <u>Schematic model showing the classes of LRR motifs in Lumican and TLR9</u>. Both lumican and TLR9 contain a mix of T, S, SDS22 and PS classes of LRRs. **A-B.** The LRR1-11 in lumican mostly consist of T and SDS22 class **(A)** whereas in TLR9 it is mostly T and irregular **(B)**.

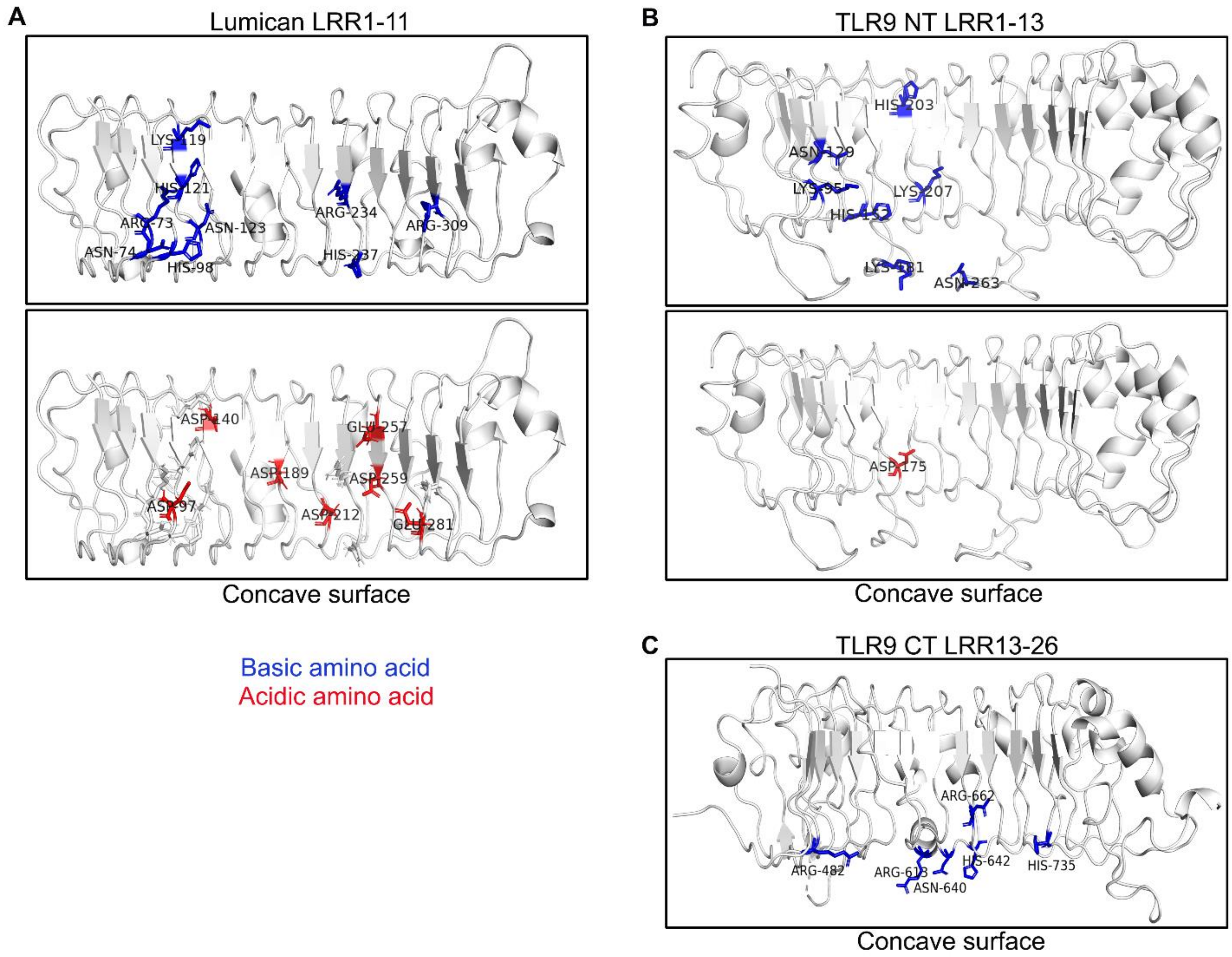

<u>Figure S3: Acidic and basic interface residues in the concave surface of Lumican and TLR9</u>. A-C. The electrostatic view shows the acidic residues (red) and basic residues (blue) within the concave surface of lumican **(A)**, TLR9 NT **(B)** and TLR9 CT **(C)**,that interact with the CpG ODN_2395.

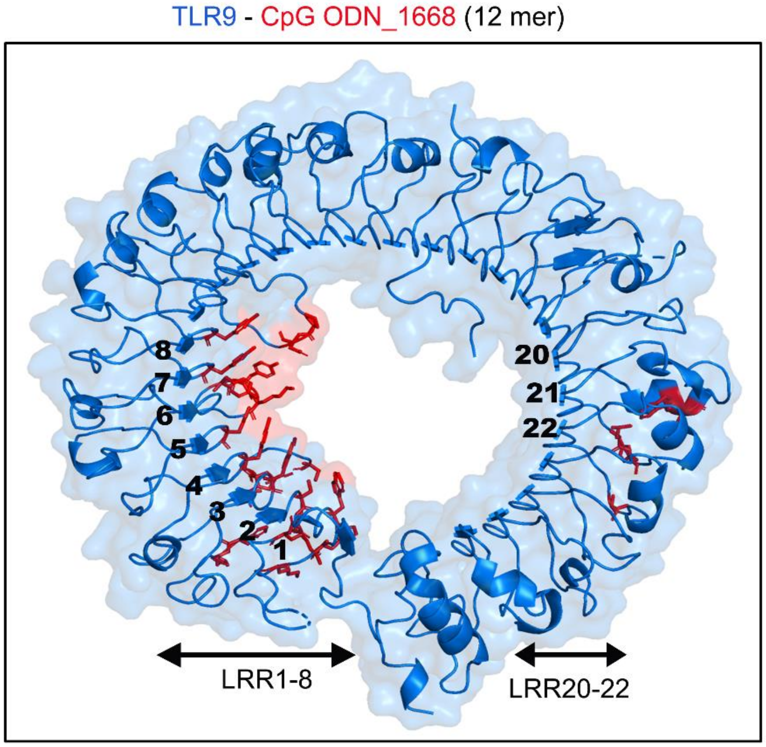

Figure S4: <u>Ribbon model showing all the TLR9 residues that interacts with the 12 mer CpG ODN_1668</u>. The model shows all the interface residues (red) in TLR9 LRR 1-8 and LRR 20-22 that interact with the shorter 12 base CpG ODN_1668.

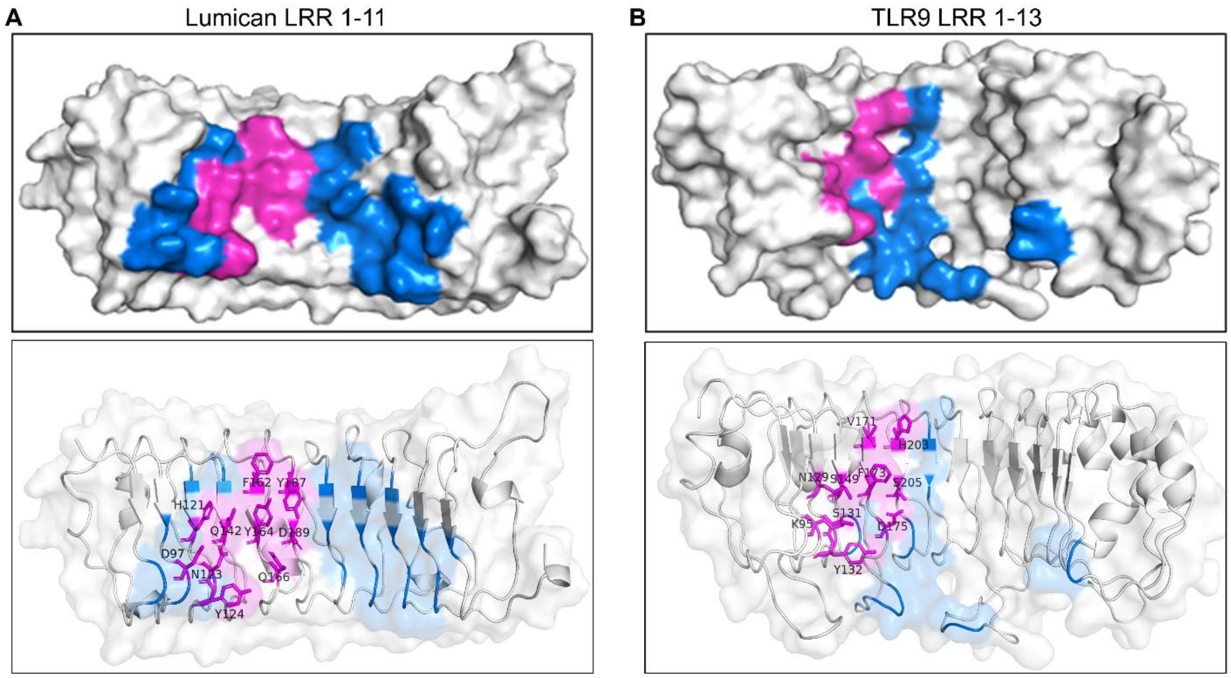

C

Lumican	TLR9	Biochemical property	Pairwise sequence alignment score	LRR #
Asp(D)97	Lys(K)95	Hydrophilic	·	2
His(H)121	Asn(N)129		:	3
Asn(N)123	Ser(S)131	Polar	:	
Tyr(Y)124	Tyr(Y)132	Aromatic	I	
Gln(Q)142	Ser(S)149	Hydrophilic	.	4
Phe(F)162	Val(V)171	Hydrophobic	.	5
Tyr(Y)164	Phe(F)173	Aromatic	:	
Gln(Q)166	Asp(D)175	Polar	.	
Tyr(Y)187	His(H)203	Both can be protonated and can form covalent bond	:	6
Asp(D)189	Ser(S)205	Hydrophilic	·	

I Identical **:** Semi-conservative · Least-conservative

<u>Figure S5: CpG DNA interacting residues in Lumican and TLR9 along with their Pairwise Sequence Alignment score</u>. A-B. Surface view showing the CpG ODN_2395 interacting

residues (blue and pink) in the concave surface of lumican LRR1-11 **(A)** and TLR9 LRR1-13 **(B)**. **C.** Table shows the list of residues (pink) with their pairwise sequence alignment scores from lumican and TLR9 Clustal Omega alignment.
